# Effect of Diet and Exercise on the Peripheral Immune System in Young Balb/c Mice

**DOI:** 10.1155/2015/458470

**Published:** 2015-11-08

**Authors:** B. E. Martínez-Carrillo, R. A. Jarillo-Luna, R. Campos-Rodríguez, R. Valdés-Ramos, V. Rivera-Aguilar

**Affiliations:** ^1^Sección de Posgrado e Investigación, Escuela Superior de Medicina, Instituto Politécnico Nacional, Plan de San Luis y Díaz Mirón, 11340 Ciudad de Mexico, DF, Mexico; ^2^Centro de Investigación y Estudios Avanzados en Ciencias de la Salud (CIEACS), Facultad de Medicina, Universidad Autónoma del Estado de México, Paseo Tollocan, Esquina Jesús Carranza, Colonia Moderna de la Cruz, 50180 Toluca, MEX, Mexico; ^3^Coordinación de Morfología, Departamento de Formación Básica Disciplinaria, Escuela Superior de Medicina, Instituto Politécnico Nacional, Plan de San Luis y Díaz Mirón, 11340 Ciudad de Mexico, DF, Mexico; ^4^Departamento de Microbiología, UBIPRO, FES-Iztacala, UNAM, Avenida de los Barrios s/n, 54090 Tlalnepantla, MEX, Mexico

## Abstract

Although diet and exercise clearly have an influence on immune function, studies are scarce on the effect caused by exercise and the consumption of a carbohydrate-rich or fat-rich diet on the peripheral immune system. The aim of the present study was to evaluate the effect of exercise and the two aforementioned unbalanced diets on young Balb/c mice, especially in relation to BMI, the level of glucose, and the percentage of lymphocyte subpopulations in peripheral blood. The changes found were then related to the synthesis of leptin and adiponectin as well as the production of oxidative stress. The increase in BMI found with the carbohydrate-rich and fat-rich diets showed correlation with the levels of leptin and adiponectin. An increase in leptin and a decrease in adiponectin directly correlated with an increase in total lymphocytes and CD4+ cells and with a decrease in B cells. The increase in leptin also correlated with an increase in CD8+ cells. Glycemia and oxidative stress increased with the two unbalanced diets, negatively affecting the proliferation of total lymphocytes and the percentage of B cells, apparently by causing alterations in proteins through carbonylation. These alterations caused by an unbalanced diet were not modified by moderate exercise.

## 1. Introduction

To maintain an optimum state of health, consumption of a balanced diet is required. Such a balance includes diverse nutrients, including proteins, carbohydrates, and lipids [1, 2]. Lipids exert a modulating effect on the function of immune cells, since fatty acids are part of their cellular composition [3]. Hence, the composition of fatty acids in the diet is likely to affect the immune response.

For this reason, it has been suggested that the content of monounsaturated fatty acids and polyunsaturated fatty acids in the diet may have an immunomodulatory effect [4], which could possibly be used in the treatment of inflammatory or autoimmune diseases [5]. Similarly, an increase in the consumption of carbohydrates modifies the number of blood cells, diminishes phagocytosis and the respiratory burst of neutrophils and macrophages, and decreases the production of proinflammatory cytokines [6–11].

The consumption of diets rich in lipids and carbohydrates increases the quantity of adipose tissue, which in turn modifies the secretion of diverse hormones, such as leptin and adiponectin (both secreted by adipose tissue). There is a direct and positive relation between the level of leptin and the quantity of adipose tissue. Contrarily, adiponectin diminishes with the increase in this tissue in obese individuals [12]. Both of these hormones have an effect on the immune response, with leptin promoting the secretion of proinflammatory cytokines [13, 14] and adiponectin inhibiting the same [15, 16].

On the other hand, moderate exercise can improve the function of the immune system, favoring a change in the immune response from Th1 to Th2. Studies have shown that moderate exercise increases* in vivo* and* in vitro* cytotoxic activity as well as the survival rate of older rats infected with influenza virus. Contrarily, strenuous or prolonged exercise boosts the production of reactive oxygen species (ROS) and thus leads to greater oxidative stress, diminished immune functions, and a rise in morbidity [17–20].

To date, there have been scarce studies on the effect caused by exercise combined with diets high in carbohydrates and lipids on the peripheral immune system. Therefore, the aim of the present study was to evaluate the effect of the consumption of carbohydrate-rich and fat-rich diets on young Balb/c mice, either sedentary or undergoing moderate exercise. The parameters measured were BMI, the level of glucose, and the population of lymphocytes in peripheral blood, and the relation between these changes and the synthesis of leptin and adiponectin, as well as the production of oxidative stress.

## 2. Methods

### 2.1. Animals

The present experimental, prospective, controlled, and randomized study was conducted with 21-day-old male Balb/c mice obtained from the bioterium of the Escuela Superior de Medicina, Instituto Politécnico Nacional. Animal care and experimental procedures were carried out in accordance with the standards of the Internal Regulation for the Use of Lab Animals of the Universidad Autónoma del Estado de México and the Animal Care and Use Committee of the Escuela Superior de Medicina, as well as the guidelines of the Mexican Secretary of Health for the Production and Care of Lab Animals (NOM-062-ZOO-1999 Ministry of Agriculture, Mexico City, Mexico). Animals were housed in individual cages during the entire experiment and food was offered* ad libitum* (from the 4th to the 12th week of life). All animals were maintained on a 12/12 h light/dark cycle.

### 2.2. Experimental Groups (Diet and Exercise)

Mice were randomly assigned to one of two experimental groups: with moderate exercise (*n* = 24) and sedentary (*n* = 24). Each group was divided into three subgroups for the administration of different diets: (1) a standard diet (control group with the Test Diet AIN-93G, Growth Purified Diet, Cat. number 57W5, with an energetic contribution of 3.97 kcal/g), (2) a carbohydrate-rich diet (CHO: DIO Rodent Purified Diet, Cat. number 58Y2, with an energetic contribution of 4.65 kcal/g), and (3) a fat-rich diet (DIO Rodent Purified Diet, Cat. number 58V8, with an energetic contribution of 3.78 kcal/g). All three diets were isocaloric and balanced in proteins (Tables 1 and 2). Three assays were performed for each group.

The mice of the exercise group underwent an adaptation period during the first week, swimming 10 min on the first day and increasing this time 5 min per day until reaching 30 min on the fifth day. During the following 8 weeks of the experiment, animals were submitted to swimming for 30 min, five days per week (Monday through Friday). Before exercising, the animals were fasted for 2 hours. Swimming was carried out in plastic tubs with lanes, placing one animal per lane to facilitate physical activity and observation. Water temperature was maintained at 37°C to avoid additional stress.

### 2.3. Quantification of the Body Mass Index (BMI)

The BMI of animals was quantified at the beginning (week 4) and at the end (week 12) of the study by using the formula BMI = mass (g)/length (cm)^2^. Length was determined by measuring the animal from the nose to the anus [21, 22]. Afterwards, the difference was calculated between the BMI of the animals at week 12 and week 4, which was recorded as the increase.

### 2.4. Collection of Biological Samples

Immediately after the last cycle of exercise (at the end of the 9th week of the experiment), the animals were anaesthetized with pentobarbital (80 mg/kg), bled by direct cardiac puncture (using a syringe with heparin), and sacrificed by cervical dislocation. From the blood samples, leukocytes were purified utilizing Ficoll-Hypaque Plus (GE Healthcare Bio-Sciences AB, Sweden). Cells were maintained in a cold RPMI-1640 medium (Sigma-Aldrich, USA) for quantification, being dyed with trypan blue and counted in a Neubauer chamber.

### 2.5. Determination of Carbonylated Proteins

Protein carbonyl groups were detected and quantified using 2,4-dinitrophenylhydrazine (DNPH) [23]. Briefly, 0.5 mL serum (1 mg protein/mL) was treated with 0.5 mL 10 mM DNPH in 2 M HCl, or with 0.5 mL 2 M HCl alone for the blank. Samples were incubated for 1 h at room temperature in the dark and then treated with 10% trichloroacetic acid and centrifuged. The pellet was washed three times in ethanol/ethyl acetate and solubilized in 1 mL of 6 M guanidine with 20 mM potassium phosphate, adjusted to pH 2.3 with trifluoroacetic acid. The resulting solution was incubated at 37°C for 15 min. The carbonyl concentration was determined from the difference in absorbance at 370 nm between DNPH-treated and HCl-treated samples, with *ε*370 = 22,000 M^−1^ cm^−1^. The carbonyl content was expressed as nanomoles of carbonyl per milligram of protein.

### 2.6. Quantification of Glucose and Hormones

Other subgroups of sedentary and exercised animals were used for obtaining peripheral blood and serum. The samples were taken immediately after exercise, or after 2 h of fasting in the sedentary group. The level of glucose was determined in peripheral blood by the GOD-PAP colorimetric method, employing the appropriate reactives (Randox, Cat. number GL2622) and a Selectra apparatus. The level of leptin and adiponectin was quantified in serum utilizing the appropriate ELISA Kit (Linco Research, Cat. number EZML-82K and EZMADO-60K, resp.) and following the manufacturer's recommendations.

### 2.7. Determination of Cell Proliferation

Once lymphocytes were obtained, a sample was placed in each well of a 12-well plate, along with 1 × 10^6^ cells/mL of RPMI-1640 medium supplemented with 5% fetal bovine serum (Invitrogen, USA; Cat. number 10091-155) and 10 *μ*L/mL penicillin/streptomycin (Sigma-Aldrich, USA; Antibac 100x, Cat. number P4333). Then, phytohaemagglutinin (PHA; Invitrogen, USA; Cat. number 10576-015) and medium were added, and the wells were incubated during 48 h in a humid atmosphere with 5% CO_2_ at 37°C. At 48 h, the supernatant was collected and the cell count was again made in a Neubauer chamber [24, 25].

### 2.8. Flow Cytometry

In each tube were placed 1 × 10^6^ cells, and then 20 *μ*L of the antibody mixture was added before incubation in the dark at 4°C for 30 min. Then, two washes were carried out, each one for 5 min each at 900 g by adding 1 mL of Hank's medium. The cell pellet was resuspended with 400 *μ*L of 1% paraformaldehyde and the samples were maintained at 4°C until reading them in the cytometer. The following monoclonal antibodies were utilized (Becton Dickinson): CD3 (FITC, Cat. number 553062), CD4 (PE, Cat. number 553730), CD8 (APC, Cat. number 553035), IgA (FITC, Cat. number 559354), and CD19 (PE, Cat. number 553786). The reading was performed on a flow cytometer (FACSCalibur, BD), counting 20,000 events per tube and utilizing Cell Quest (Becton Dickinson). Data were processed with FACS Diva 6.0 software (Becton Dickinson).

### 2.9. Statistical Analysis

Data are presented as the mean ± SD. Comparison between more than two groups was performed by two-way ANOVA. If a significant main effect or association was identified, the means of the respective groups were compared using the Bonferroni *t*-test. Correlations between variables were established with the Pearson Product Moment Correlation. In all cases, a *p* value < 0.05 was considered significant. All analyses and graphics were performed with SigmaPlot software 11.0 (SPSS Inc.).

## 3. Results

### 3.1. Diet and Exercise Modified BMI

**Figure 1:**
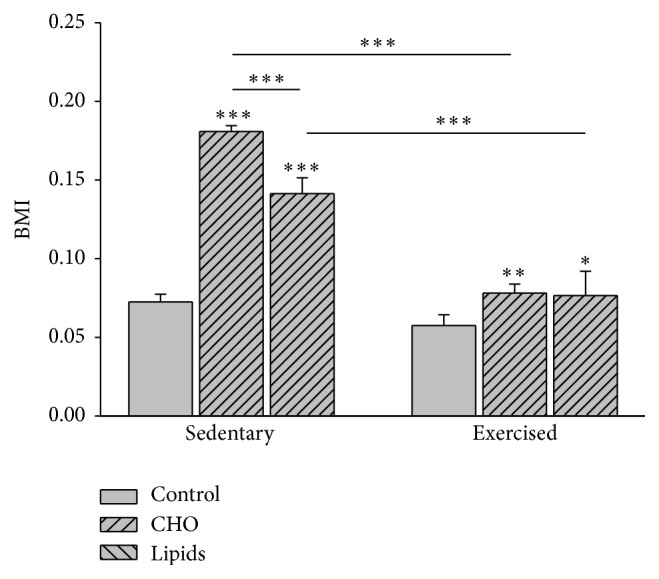
Body mass index (BMI). The BMI is determined by the formula BMI = mass (g)/length  (cm)^2^. Data indicate that the BMI increased during the experiment for all subgroups [(BMI at week 12) − (BMI at week 4)]. In the sedentary and exercised mice, both unbalanced diets (carbohydrate-rich and fat-rich) led to a greater increase in BMI than the standard (balanced) diet. Although the increase in BMI was similar for each diet when comparing the exercised and sedentary groups, it was indeed significantly lower in the exercised group (Bonferroni test: ^*∗∗∗*^ *p* < 0.001 for the carbohydrate-rich diet; ^*∗∗*^ *p* < 0.01 for the fat-rich diet; ^*∗*^ *p* < 0.05 for the standard diet). Data are expressed as the mean ± SD (*n* = 6 per treatment group).

Diet (*F* = 170.2; *p* < 0.001) and exercise (*F* = 435.6; *p* < 0.001) had an effect on BMI. Sedentary and exercised mice fed carbohydrate-rich and fat-rich diets showed a greater increase in BMI than those with the standard diet. Compared to the standard diet, the carbohydrate-rich diet produced the greatest increase (*p* < 0.001 for the sedentary group; *p* = 0.004 for the exercised group) and the fat-rich diet a lesser increase (*p* = 0.01 for the exercised group). Compared to exercised animals, sedentary animals with the carbohydrate-rich or fat-rich diet showed a greater increase in BMI (*p* < 0.001 in both cases; Figure 1). On the other hand, the sedentary and exercised groups with the standard diet showed a similar increase in BMI.

### 3.2. Diet and Exercise Modified Glycemia

**Figure 2:**
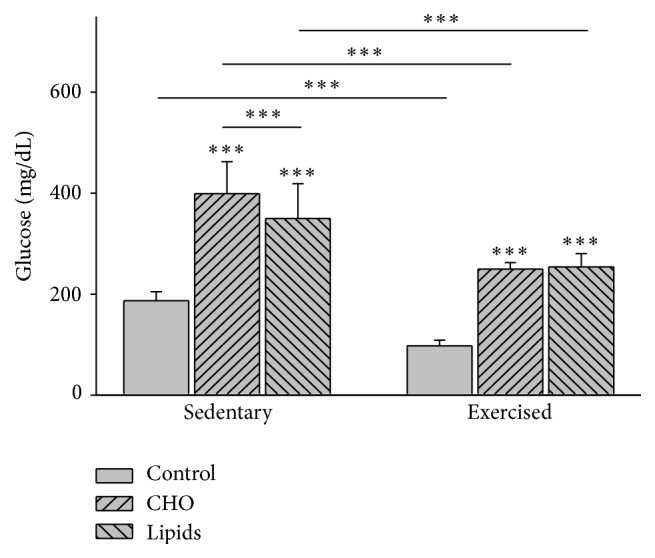
Plasma glucose. Glucose was determined by the colorimetric method at the end of the experiment. In the sedentary and exercised mice, the carbohydrate-rich and fat-rich diets caused a higher level of glycemia than that found in animals with the standard diet. Compared to sedentary mice, exercised animals had a lower level of glycemia with all diets (Bonferroni test ^*∗∗∗*^ *p* < 0.001). Data are expressed as the mean ± SD (*n* = 6 per treatment group).

Given the relation between the quantity of adipose tissue and glycemia, the concentration of plasmatic glucose was evaluated. Diet (*F* = 94, *p* < 0.001) and exercise (*F* = 88.7, *p* < 0.001) had an effect on glucose levels. Compared to the subgroups with the standard diet, the sedentary and exercised mice fed with the carbohydrate-rich or fat-rich diet showed greater levels of glucose (*p* < 0.001). Within the sedentary group, a higher glucose level was found in animals fed with the carbohydrate-rich diet than the fat-rich diet (*p* < 0.001). Within the exercised group, there was no difference in this parameter between those fed with the carbohydrate-rich and fat-rich diets. On the other hand, a lower level of glucose was found in the exercised versus sedentary animals for all three dietary subgroups (*p* < 0.001; Figure 2).

### 3.3. Diet and Exercise Modified the Level of Serum Leptin

**Figure 3:**
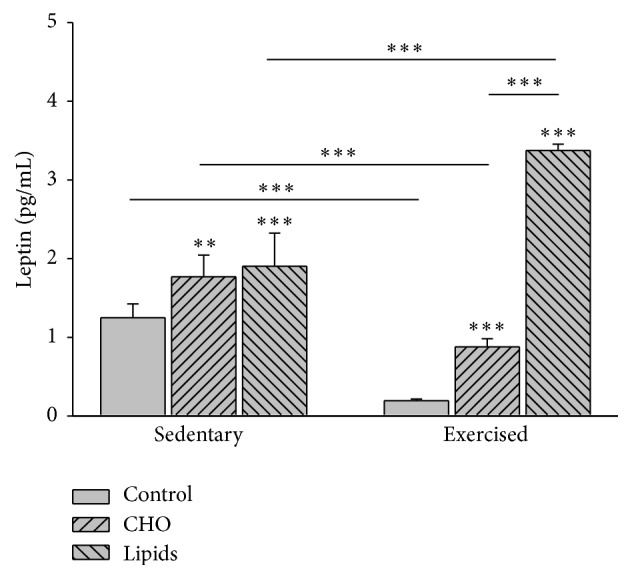
Serum leptin. The sedentary and exercised mice showed a higher plasmatic level of leptin with carbohydrate-rich and fat-rich diets compared to animals with the standard diet. For exercised mice, the increase in leptin was greater with a fat-rich than carbohydrate-rich diet. Compared to sedentary mice, exercised animals had a lower level of leptin when fed with a standard or carbohydrate-rich diet, but they had a higher level of this hormone when given a fat-rich diet. Determination was made by ELISA at the end of the experiment (Bonferroni test ^*∗∗∗*^ *p* < 0.001; ^*∗∗*^ *p* < 0.01). Data are expressed as the mean ± SD (*n* = 6 per treatment group).

Diet (*F* = 229.4; *p* < 0.001) and exercise (*F* = 4.4; *p* < 0.04) influenced the plasmatic levels of leptin. Within the sedentary group, a higher level of leptin was found in the subgroups fed with the carbohydrate-rich (*p* = 0.006) or fat-rich (*p* < 0.001) diet than the animals with the standard diet. The same pattern was found within the exercised group, with a greater increase in leptin for the animals with the fat-rich than carbohydrate-rich diet (*p* < 0.001). When comparing exercised and sedentary mice, the former had a lower level of leptin when fed with the standard or carbohydrate-rich diet but had a higher level of this hormone when given the fat-rich diet (*p* < 0.001; Figure 3).

### 3.4. Diet but Not Exercise Modified the Level of Serum Adiponectin

**Figure 4:**
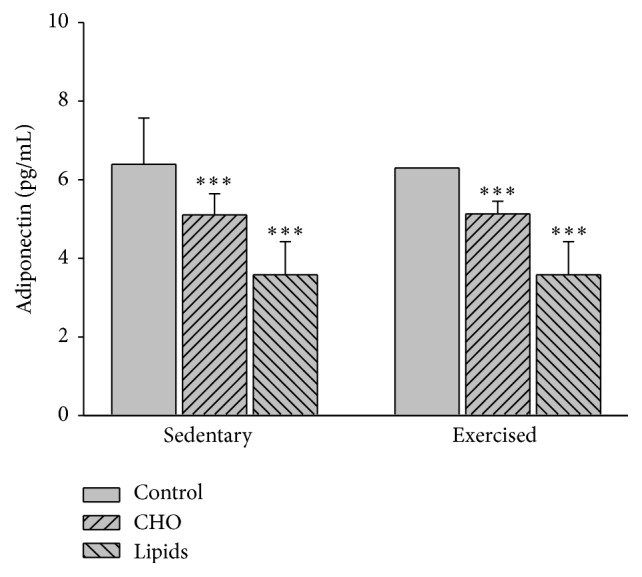
Serum adiponectin. In sedentary and exercised groups, the level of adiponectin was lower in mice with the carbohydrate-rich than standard diet and lower in animals with the fat-rich than the carbohydrate-rich diet. No differences were observed between the sedentary and exercised animals. Determination was made by ELISA at the end of the experiment (Bonferroni test ^*∗∗∗*^ *p* < 0.001). Data are expressed as the mean ± SD (*n* = 6 per treatment group).

Diet (*F* = 43.1; *p* < 0.001) but not exercise (*F* = 0.007; *p* = 0.93) influenced the level of adiponectin. Whereas no difference was found between exercised and sedentary mice, within each of these groups, there was a lower adiponectin level in animals fed with the fat-rich versus carbohydrate-rich diet (*p* < 0.001), as well as those fed with the carbohydrate-rich versus standard diet (*p* < 0.001; Figure 4).

### 3.5. Correlation between BMI, Hormones, and Glucose

There was a positive correlation between the increase in BMI and the concentration of glucose in sedentary (*r* = 0.8; *p* < 0.001) and exercised mice (*r* = 0.71; *p* < 0.001). On the other hand, the increase in BMI correlated positively with the level of leptin in the sedentary group (*r* = 0.56; *p* = 0.01) but not in the exercised group. The level of adiponectin showed a negative correlation with the increase in BMI in sedentary (*r* = −0.48; *p* = 0.04) and exercised mice (*r* = −0.64; *p* = 0.003).

### 3.6. Diet and Exercise Modified Cell Proliferation

**Figure 5:**
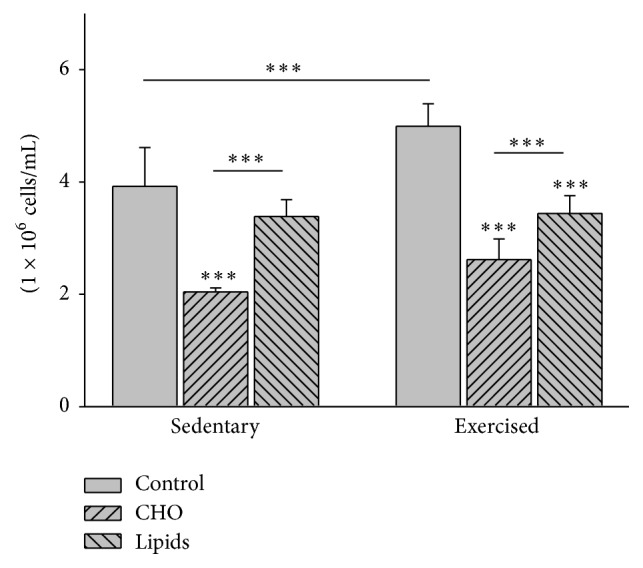
Proliferation of lymphocytes in peripheral blood. Postculture quantification of lymphocytes was performed in a Neubauer chamber. Whiting the sedentary and exercised groups, there was a lower level of cell proliferation in mice with the modified diets than the standard diet, and a lower level in animals with the carbohydrate-rich than fat-rich diet. Comparing the sedentary and exercised mice, there were no differences in this parameter between the groups with modified diets. There was a lower level of this parameter in the sedentary than exercised group with a standard diet (Bonferroni test ^*∗∗∗*^ *p* < 0.001). Data are expressed as the mean ± SD (*n* = 6 per treatment group).

After 48 h of culturing lymphocytes and stimulating them with PHA, cell counts showed that diet (*F* = 112.7; *p* < 0.001) and exercise (*F* = 23.9; *p* < 0.001) both influenced cell proliferation. Within the sedentary and the exercised groups, there was less cell proliferation of lymphocytes for the animals fed with the carbohydrate-rich than the fat-rich (*p* < 0.001 and *p* = 0.003, resp.) or standard diet (*p* < 0.001 for both subgroups). When comparing sedentary and exercised mice, the respective subgroups with the carbohydrate-rich or fat-rich diet showed similar cell proliferation of lymphocytes. However, with a standard diet, the value of this parameter was lower in the sedentary than exercised group (*p* < 0.001; Figure 5).

### 3.7. Diet but Not Exercise Modified the Level of Carbonylated Proteins in Lymphocytes

**Figure 6:**
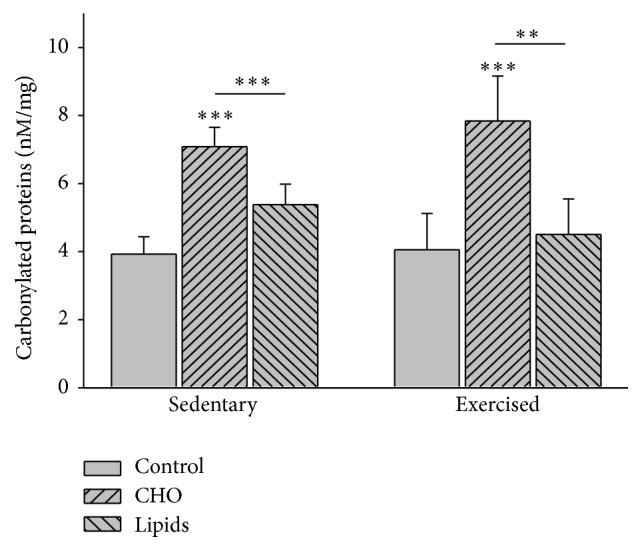
Carbonylated proteins in lymphocytes of peripheral blood. Protein carbonyl groups were detected and quantified using 2,4-dinitrophenylhydrazine (DNPH). Regarding the sedentary and exercised mice, higher concentrations of carbonylated proteins were found in groups with the modified diets than the standard diet, and the highest levels existed in the animals with carbohydrate-rich diet. No differences were observed when comparing the sedentary groups with the exercised groups (Bonferroni test ^*∗∗∗*^ *p* < 0.001, ^*∗∗*^ *p* < 0.01). Data are expressed as the mean ± SD (*n* = 6 per treatment group).

Since reactive oxygen species (ROS) are produced by normal cell metabolism and moderate exercise is capable of mitigating the deleterious effects of these free radicals, carbonylated proteins in lymphocytes were quantified to evaluate oxidative stress in these cells. Diet (*F* = 61; *p* < 0.001) but not exercise (*F* = 0.22; *p* = 0.641) modified the quantity of carbonylated proteins in lymphocytes. Within the sedentary group, there was a higher concentration of carbonylated proteins in animals fed with the carbohydrate-rich than the standard (*p* < 0.001) or fat-rich diet (*p* = 0.007). Within the exercised group, a similar result was found (*p* < 0.001 in both cases). However, no difference was found in this parameter when comparing sedentary and exercised mice in relation to any given dietary regimen (Figure 6).

### 3.8. Correlation between Glucose, Cell Proliferation, Carbonylated Proteins, and Hormones

The glucose level correlated positively with the concentration of carbonylated proteins and negatively with the cell proliferation of lymphocytes in sedentary (*r* = 0.75, *p* < 0.001 and *r* = −0.6, *p* < 0.001, resp.) and exercised mice (*r* = 0.4, *p* = 0.02 and *r* = −0.8, *p* < 0.001, resp.). Likewise, the concentration of carbonylated proteins correlated negatively with the cell proliferation of lymphocytes in sedentary (*r* = −0.8; *p* < 0.001) and exercised (*r* = −0.6; *p* = 0.001) animals. Finally, a positive correlation was observed between the cell proliferation of lymphocytes and the level of adiponectin in exercised (*r* = 0.5; *p* = 0.009) but not sedentary animals, and no correlation was found between the cell proliferation of lymphocytes and the level of leptin.

### 3.9. Diet/Exercise with regard to the Percentage of CD19, CD3+, and CD4+ Lymphocytes

**Figure 7:**
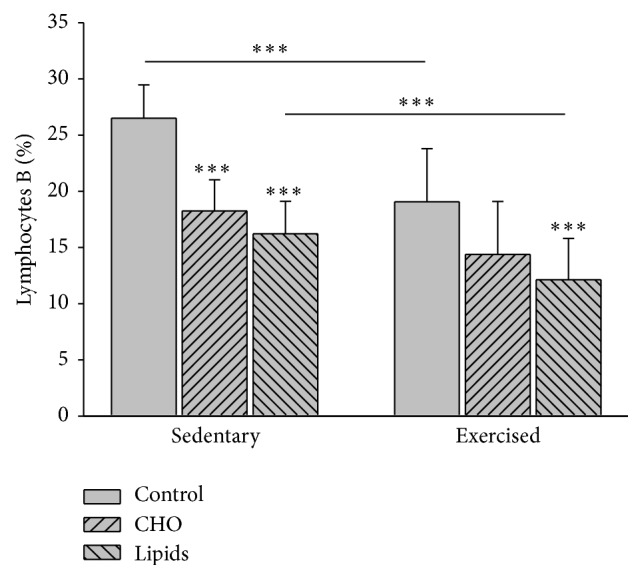
B lymphocytes in peripheral blood. Cells were quantified by flow cytometry. The pattern is clearly similar for sedentary and exercised mice, with a lower percentage of B lymphocytes in the animals with modified diets than those with a standard diet. However, in the exercised mice, the lower value for the carbohydrate-rich diet than the standard diet did not represent a significant difference. The sedentary mice had a higher percentage of B lymphocytes in the groups with the standard and fat-rich diet than the respective groups of the exercised animals (Bonferroni test ^*∗∗∗*^ *p* < 0.001). Data are expressed as the mean ± SD (*n* = 6 per treatment group).

Diet (*F* = 23.2; *p* < 0.001) and exercise (*F* = 22.7; *p* < 0.001) modified the percentage of B lymphocytes (CD19). Within the sedentary group, the carbohydrate-rich and fat-rich diets both caused a decrease in the percentage of B lymphocytes compared to the standard diet (*p* < 0.001). There was no difference in this parameter between the two unbalanced diets. Within the exercised group, only the fat-rich diet caused a decrease in the percentage of B lymphocytes compared to the standard diet (*p* < 0.001). Comparing exercised and sedentary mice, the former showed a lower percentage of B cells with a standard or fat-rich diet (*p* < 0.001), but similar percentages were found for both groups with regard to the carbohydrate-rich diet (Figure 7).

**Figure 8:**
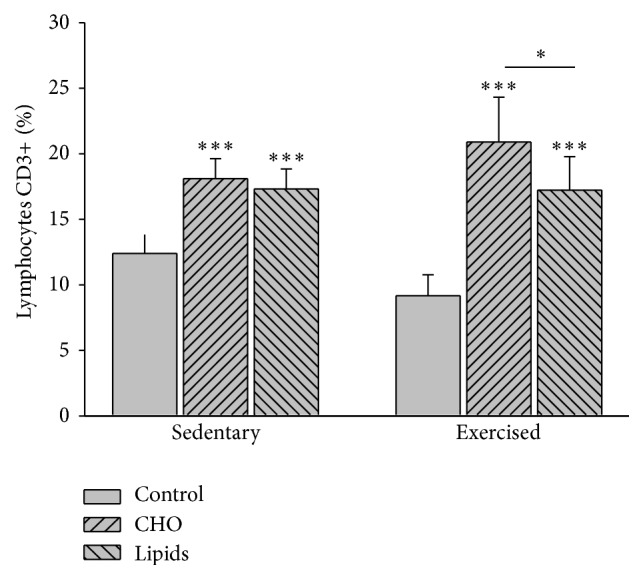
CD3+ T lymphocytes in peripheral blood. Cells were quantified by flow cytometry. In the sedentary and exercised mice, there was a higher percentage of CD3+ lymphocytes in the animals with a modified diet than in those with a standard diet. For the exercised mice, the carbohydrate-rich diet produced a higher percentage of these cells than the fat-rich diet. There was no difference in this sense among the sedentary mice. No differences were found between the sedentary and exercised mice (Bonferroni test ^*∗∗∗*^ *p* < 0.001). Data are expressed as the mean ± SD (*n* = 6 per treatment group).

Diet (*F* = 68.8; <0.001) but not exercise (*F* = 0.16; *p* = 0.7) had an effect on the percentage of CD3+ T lymphocytes. Within the sedentary and exercised groups, the percentage of CD3+ lymphocytes was greater in animals fed with the carbohydrate-rich and fat-rich diets than those with the standard diet (*p* < 0.001 in both cases). For the exercised mice, there was a greater percentage of these T lymphocytes in the group with a carbohydrate-rich than fat-rich diet (*p* = 0.04). Contrarily, there was no difference between the two unbalanced diets for the sedentary mice. No differences were found for any given diet between exercised and sedentary animals (Figure 8).

**Figure 9:**
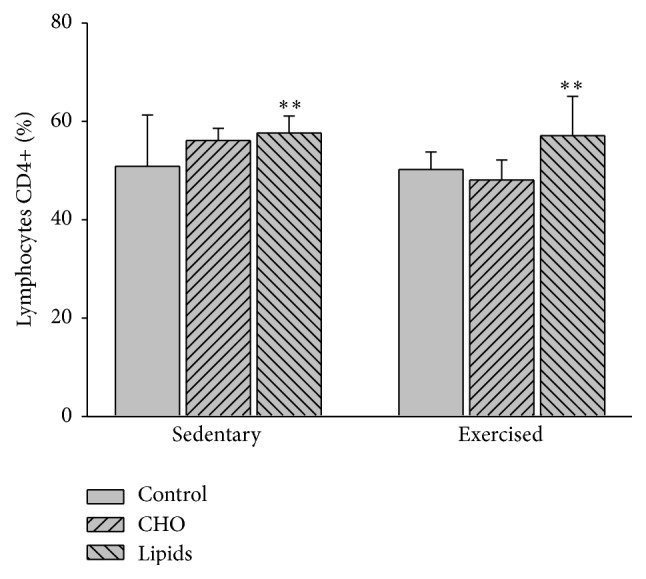
CD4+ lymphocytes in peripheral blood. Cells were quantified by flow cytometry. Whiting the sedentary and exercised groups, only the fat-rich diet was significantly different than the respective group with a standard diet. No differences were observed between the sedentary and exercised mice (Bonferroni test ^*∗∗*^ *p* < 0.01). Data are expressed as the mean ± SD (*n* = 6 per treatment group).

Diet (*F* = 5.6; *p* < 0.007) but not exercise (*F* = 3.1; *p* = 0.08) had an effect on the percentage of CD4+ lymphocytes. Within the sedentary and exercised groups, only the fat-rich diet showed a higher percentage of CD4+ cells than the standard diet (*p* = 0.008; Figure 9).

**Figure 10:**
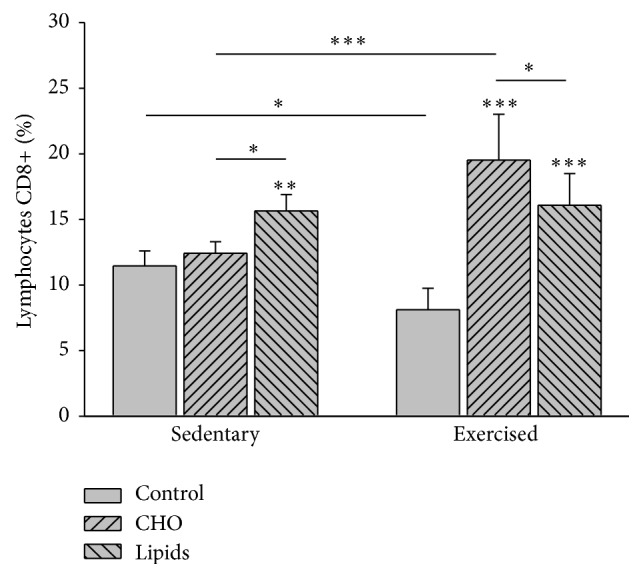
CD8+ lymphocytes in peripheral blood. Cells were quantified by flow cytometry. For the sedentary mice, animals with the fat-rich diet showed a higher percentage of cells than those with the standard or carbohydrate-rich diet. For the exercised groups, animals with the carbohydrate-rich and fat-rich diet showed a higher percentage of cells than those with the standard diet. This increase was greater in the exercised animals with a carbohydrate-rich versus fat-rich diet (Bonferroni test ^*∗∗∗*^ *p* < 0.001, ^*∗∗*^ *p* < 0.01, and ^*∗*^ *p* < 0.05). Data are expressed as the mean ± SD (*n* = 6 per treatment group).

Diet (*F* = 49.6; *p* < 0.001) and exercise (*F* = 5.8; *p* = 0.02) modified the percentage of CD8+ lymphocytes. Within the sedentary group, the fat-rich diet produced a greater percentage of CD8+ cells than the standard (*p* = 0.002) and carbohydrate-rich diet (*p* = 0.04). Within the exercised group, both the fat-rich and carbohydrate-rich diets led to an increase in this parameter compared to the standard diet (*p* < 0.001). This increase was greater in the exercised animals with a carbohydrate-rich than fat-rich diet (*p* = 0.02). Comparing sedentary and exercised animals, the former had a higher percentage of CD8+ cells when fed with the standard diet (*p* = 0.03), while the latter had a higher percentage of these cells with the carbohydrate-rich diet (*p* < 0.001; Figure 10).

### 3.10. Correlation between the Distinct Types of Lymphocytes and Measured Hormones

The percentage of B lymphocytes correlated negatively with the level of leptin but positively with the level of adiponectin in the sedentary (*r* = −0.7, *p* < 0.001 and *r* = 0.5, *p* = 0.002, resp.) and exercised groups (*r* = −0.4, *p* = 0.01 and *r* = −0.05, *p* = 0.003, resp.). The percentage of CD3+ lymphocytes showed a positive correlation with the level of leptin in the sedentary (*r* = 0.5; *p* = 0.003), but not exercised group, and a negative correlation with the level of adiponectin in the sedentary (*r* = −0.5; *p* = 0.007) and exercised animals (*r* = −0.4; *p* = 0.02).

The percentage of CD4+ lymphocytes had a positive correlation with the level of leptin in sedentary (*r* = 0.04; *p* = 0.03) and exercised mice (*r* = 0.5; *p* = 0.007), and a negative correlation with adiponectin in the exercised animals (*r* = −0.5; *p* = 0.003).

The percentage of CD8+ lymphocytes had a positive correlation with leptin in sedentary mice (*r* = 0.4, *p* = 0.04) but no such correlation was found with exercised animals (*r* = 0.36; *p* = 0.06). These same lymphocytes showed a negative correlation with adiponectin in both the sedentary (*r* = −0.7; *p* < 0.001) and exercised groups (*r* = −0.4; *p* = 0.01).

### 3.11. Correlation between Oxidative Stress and the Distinct Types of Lymphocytes

There was a negative correlation between the percentage of CD19 lymphocytes and the concentration of carbonylated proteins in the sedentary group (*r* = −0.6; *p* = 0.002), but no such correlation was found in the exercised group. On the other hand, the percentage of CD3+ lymphocytes correlated positively with the concentration of carbonylated proteins in both the sedentary (*r* = 0.86; *p* < 0.001) and exercised groups (*r* = 0.72; *p* < 0.001).

## 4. Discussion

The present results provide evidence that the consumption of unbalanced diets increases glycemia and oxidative damage to lymphocytes in peripheral blood, affecting the proliferation of these cells* in vitro* and modifying the percentages of T and B lymphocytes* in vivo*. The secretion of leptin and adiponectin was also affected by unbalanced diets.

### 4.1. Influence of Diet on BMI, Glycemia, and Hormones

In the absence of exercise, the consumption of a carbohydrate-rich or fat-rich diet was directly related to a greater BMI, which in turn showed an association with the hyperglycemia observed in these animals. It is noteworthy that the sedentary animals fed with the standard diet (and after 2 h of fasting) showed higher glycemia than that reported for Balb/c mice after four hours of fasting [26]. This difference in results could be due to less hours of fasting in the present study or to the different method of quantification used.

The increase in BMI was accompanied by a rise in the level of leptin and a reduction in the level of adiponectin. The carbohydrate-rich and fat-rich diets provoked these changes in spite of being isocaloric. The carbohydrate-rich diet induced the greatest increase in the BMI.

Although in the present study the quantity of adipose tissue was not quantified, it was indeed observed qualitatively. Accordingly, there was an increase in the quantity of the abdominal adipose tissue in the sedentary group with either of the two unbalanced diets. We therefore consider that the greater BMI in these sedentary animals is an indicator of an increase in adipose tissue. The positive correlation between a greater BMI and an increase in adipose tissue has been previously reported in mice [27, 28] and rats [22].

The level of leptin rose in direct relation to the increase in BMI in the sedentary group. Considering that a greater BMI is mostly due to an increase in adipose tissue, the present results coincide with previous reports in the sense that levels of leptin are directly related to the quantity of this tissue [29–32]. On the other hand, the level of serum adiponectin was negatively correlated with the greater BMI observed in animals with a carbohydrate-rich or fat-rich diet (the greater the BMI, the lower the levels of adiponectin). Hence, the decrease in the level of adiponectin found herein is in accordance with multiple reports of the inverse relation between the quantity of adipose tissue and the secretion of adiponectin [33–36].

Within the exercised group, there was a positive correlation between the increase in both BMI and glycemia accompanying the carbohydrate-rich and fat-rich diet (compared to the standard diet). The sedentary mice showed a greater increase in BMI and glycemia than the exercised animals with the same diets, which is in agreement with the previous reports [37–39].

With respect to hormones, although the level of leptin in animals with unbalanced diets was higher than in those with the standard diet, there was no correlation of this parameter with the increase in BMI. Other mechanisms could have conditioned the smaller increase in this hormone compared to BMI, including changes in the secretion of insulin during exercise [40]. The greater level of leptin accompanying a fat-rich diet can be explained by at least two factors. The consumption of fat leads to an increase in the secretion of glucocorticoids [41–43] and the consumption of n-3 fatty acids increases the secretion of leptin [44, 45].

On the other hand, the low level of leptin in mice fed with the carbohydrate-rich diet could make these animals prone to obesity. The fact that this did not occur is perhaps due to the greater sensitivity in the brain to this hormone during exercise, as previously reported [46, 47].

In the present study, the level of adiponectin was unchanged by exercise, which is in agreement with some studies [48, 49] but not with others reporting that this hormone increases with exercise [50, 51].

### 4.2. The Unbalanced Diets Decreased Cell Proliferation

Within the exercised group, the fat-rich diet diminished the proliferation of peripheral blood lymphocytes stimulated with phytohaemagglutinin (PHA), which is in agreement with other studies [50, 52]. The carbohydrate-rich diet diminished the proliferation of lymphocytes in both sedentary and exercised animals, which other authors have also reported [6–10]. Diverse studies show that exercise influences lymphocyte proliferation, such as the report on an increase in the proliferation of these cells observed in trained animals submitted to swimming [53]. Overall, it seems clear that the influence of diet predominated over that of exercise in the present study.

The mechanism by which carbohydrates and lipids reduce lymphocyte proliferation is unknown. The current results show that the change in the proliferation of these cells is not associated with the levels of leptin or adiponectin in the sedentary group. In the exercised group, the proliferation of lymphocytes was directly proportional the level of adiponectin. Hence, the positive effects of leptin on the proliferation of B and T lymphocytes [34, 54–58] and the negative effects of adiponectin on this same cell proliferation [34, 53, 59] were not evident in the current results. This discrepancy with previous reports is possibly due to the present protocol, in which lymphocytes were not submitted to the effect of adiponectin in culture. Although lymphocytes secrete adiponectin [60, 61], the* in vitro* conditions of the present study may not have been adequate for the synthesis of this hormone.

Immune cells, including B and T lymphocytes, express receptors for leptin and adiponectin [34, 62, 63]. However, it is known that with an excess of adipose tissue the expression of these receptors is diminished [63, 64], which could explain why the autocrine activity of adiponectin was not evident.

Since the activity of these hormones apparently did not cause the decrease in lymphocyte cell proliferation, we must consider other possibilities. Based on the current results, this decrease was directly related to alterations in proteins caused by hyperglycemia. It is known that the glycation of proteins occurs in the presence of very high levels of glucose in the blood [65], causing structural and functional modifications of proteins by free radicals of oxygen. This could have induced the decrease in lymphocyte cell proliferation found herein. A relation between hyperglycemia, higher levels of carbonyl groups, and decreased lymphocyte proliferation was found in sedentary and exercised animals of the present study.

High levels of carbonylated proteins have been detected in diabetic patients as a product of glycol oxidation [66]. The accumulation of many damaged proteins triggers mechanisms that block cell division [67]. This could explain the mechanism by which the cells with a greater quantity of carbonylated proteins in the present study showed lower proliferation in culture.

Overall, it can be said that hyperglycemia, caused by the consumption of unbalanced diets, whether in the absence or in the presence of exercise, maintains a state of chronic oxidative stress that is capable of damaging multiple molecules of biological importance. Hence, diets with the capacity of elevating the level of glucose in the blood participate in an increase in oxidative stress, which leads to a decrease in the production of immune cells.

### 4.3. Effects of Diet and Exercise on Lymphocyte Subpopulations

The carbohydrate-rich and fat-rich diet increased the percentage of CD3+ lymphocytes in the sedentary and exercised groups. On the other hand, although there was a reduced percentage of B (CD19+) lymphocytes with both unbalanced diets in the sedentary group, this decrease was only evident with the fat-rich diet in the exercised group. This reduction of B cells associated with a fat-rich diet (i.e., rich in saturated fatty acids) was found in another study reporting that this diet also led to an increase in the number of T cells [68].

Likewise, the present study found an increase in the subpopulations of T lymphocytes (e.g., the percentages of CD4+ and CD8+ cells) in the animals (both sedentary and exercised) with a fat-rich diet. The percentage of CD8+ lymphocytes also increased in exercised animals with a carbohydrate-rich diet. The increase in total T lymphocytes was in direct relation to the level of leptin in sedentary mice, and in inverse relation to the level of adiponectin in both sedentary and exercised animals.

Leptin has a positive effect on the quantity of CD4+ cells by acting on virgin CD4+ cells to favor their differentiation to the CD4+ Th1 phenotype and to thus diminish the proliferation of CD4+ memory cells and Treg cells [13, 34, 62, 69]. The current results show that the level of leptin is positively correlated with the percentage of CD4+ lymphocytes in sedentary and exercised animals (considering all diets), and with the percentage of CD8+ lymphocytes in sedentary animals fed with the unbalanced diets. There are reports that leptin has a direct stimulating effect on the proliferation of CD8+ cells [70, 71] or an indirect stimulating effect through the function of dendritic cells [72]. This stimulation of CD8+ cells by leptins seems to be confirmed by the present results.

On the other hand, adiponectin reportedly has the opposite effect, increasing Treg lymphocytes and decreasing the proliferation of CD4+ and CD8+ cells [34, 73, 74]. The information in the literature on the relation of this hormone to CD8+ lymphocytes is not completely clear, as there are also reports that under different experimental conditions there is a positive correlation between adiponectin and these subpopulations of T lymphocytes [75, 76]. The current results show that a decrease in the level of adiponectin correlates with an increase in total T cells and in the percentage of CD4+ and CD8+ cells, corroborating the negative effect of this hormone on T lymphocytes.

In the present study, the decrease in B lymphocytes that took place with the two unbalanced diets showed a positive correlation with the decrease in adiponectin and a negative correlation with the increase in leptin. The information in the literature on the effects of leptin and adiponectin on B lymphocytes is contradictory. Some studies report that leptin inhibits apoptosis of these immune cells in the spleen [57, 58], while others have found that this hormone has no influence on B cells [77] or that it inhibits the development of B cells [59].

Additionally, the lymphocyte populations must have been influenced by hyperglycemia. Mouse T and B lymphocytes reportedly show a decrease in proliferation when cultivated in the presence of a high concentration of glucose [19]. However, only B lymphocytes were negatively affected by the high concentration of glucose in the two unbalanced diets of the present study. Hence, a direct relation was found between high glucose levels, an increase in carbonylated proteins, and a decrease in the percentage of B lymphocytes. These results coincide with a previous report that oxidative stress caused by hyperglycemia affects the proliferation of T and B lymphocytes [78].

We can postulate that oxidative stress may have increased the apoptosis of B cells, as has been reported [79], or that hyperglycemia diminished the production of cytokines, also previously reported [80]. On the other hand, the lower percentage of B lymphocytes in the exercised animals of the present study (regardless of diet) could also have been due to the redistribution of lymphocytes to the blood and lymphoid organs, which has been reported to occur during exercise [81, 82]. The decrease in B cells may reflect the negative impact of exercise on some aspects of the immune response [83].

Regarding the subpopulations of T cells, in mice fed with the fat-rich diet no relation was found between glycemia and the increase in CD4+ lymphocytes. Perhaps this increase resulted from a differential influence on the proliferation of T lymphocyte subtypes by the quality of fatty acids. Different types of fatty acids may change the fluidity of the T cell membrane by inhibiting enzymes bound to it or inhibiting cell viability and DNA synthesis [52, 84–88]. Contrary to CD4+ lymphocytes, the percentage of CD8+ cells had a positive correlation with glucose and carbonylated proteins in the exercised group, meaning that this lymphocyte subtype is sensitive to the activating effect of ROS [89].

According to the present study, exercise modified the effects of the carbohydrate-rich and fat-rich diets in relation to CD8+ but not CD4+ cells, which is in accordance with some studies [90, 91]. However, other studies have reported that exercise decreases [92, 93] or increases both of these subtypes [24], and, in the same sense, that exercise has little effect on the CD4/CD8 ratio [94]. The increase in CD8+ lymphocytes found presently in the exercised group was accompanied by a rise in the level of leptin. Although this apparent association did not reach the level of significance (possibly due to the sample size), we consider that leptin contributed to the changes observed in these lymphocytes, although other factors related to exercise may also have played a role, such as the mobilization of lymphocytes to the blood stream [25, 95]

Additionally, the consumption of carbohydrates during exercise generally has a positive influence on the immune response [96]. This could explain the greater percentage of CD8+ cells observed presently with the carbohydrate-rich diet in exercised animals, although we did not find any reports on a possible relation between this diet and CD8+ lymphocytes.

In summary, the consumption of unbalanced diets by young Balb/c mice caused a greater BMI than that found in animals with a standard (balanced) diet. The carbohydrate-rich and fat-rich diets did not result in obesity, apparently because these unbalanced diets were isocaloric and Balb/c mice are resistant to diets that induce obesity. A qualitative analysis showed that the greater BMI found with the unbalanced diets was accompanied by an accumulation of visceral adipose tissue. Hence, an increase in adipose tissue in sedentary animals was negatively correlated with a decrease in adiponectin and positively correlated with an increase in leptin, in accordance with previous studies. In the exercised group, other hormonal modifications could have conditioned the rise in the level of leptin, such as a decrease in insulin secretion (although this parameter was not measured).

It is clear from these results that diet was the determining factor in the changes found presently in leptin and adiponectin levels, with exercise showing a less prominent effect. In cell cultures, the stimulating effect of leptin and inhibiting effect of adiponectin on lymphocyte proliferation were not evident, possibly due to the absence of adipocytokines in the culture medium and the absence of the autocrine effect of the same.

Although the results of the present study in relation to lymphocyte subpopulations are not completely clear, it appears that there was a positive effect of leptin on the proliferation of total lymphocytes, and in particular on CD4+ cells. Likewise, the results seem to indicate a negative influence of adiponectin on the populations of total lymphocytes, and in particular on CD4+ cells. Moreover, this hormone apparently had a positive effect on the population of CD8+ cells. The effects observed on B cells contradict the reports in the literature on the influence of adipocytokines on this cell population. The unbalanced diets increased glycemia and oxidative stress that apparently induced alterations in proteins by carbonylation, thus negatively affecting the proliferation of the total population of lymphocytes as well as the percentage of B cells. These alterations were not modified with exercise.

Overall, we conclude that the type of diet consumed had important implications for the peripheral immune system in Balb/c mice. The administration of carbohydrate-rich and fat-rich diets influenced the secretion of leptin and adiponectin, increasing the presence of oxidative stress and therefore cellular damage. Even though the mice were very young, these negative influences on peripheral immunity resulting from unbalance diets were not importantly modified by exercise.

## Figures and Tables

**Table 1:** Nutrimental contribution of the diets fed to mice (%).

	AIN-93G	58Y2 (CHO)	58V8 (lipids)
CHO			
Starch	39.74	0.00	0.00
Maltodextrin	13.20	33.17	20.13
Sucrose	10.00	33.18	20.13
Total	**62.94**	**66.35**	**40.26**
Lipids			
Soybean oil	7.01	2.37	2.91
Lard	0.00	1.93	20.69
Total	**7.01**	**4.30**	**23.60**
Protein			
Casein	20.00	18.95	23.30
Total macronutrients	**89.96**	**89.60**	**87.16**
Cellulose	0.00	4.73	5.83
Vitamins and minerals	10.00	5.67	7.01

Total	100	100	100

**Table 2:** Lipid composition of the diets fed to mice.

	AIN-93G	58Y2 (CHO)	58V8 (Lipids)
Saturated fatty acids	15.5^**∗∗**^	26.5^**∗∗**^	38.30^**∗∗**^
Monounsaturated fatty acids	23.5^**∗∗**^	30.20^**∗∗**^	39.50^**∗∗**^
Polyunsaturated fatty acids	60.9^**∗**^	43.20^**∗∗**^	22.20^**∗∗**^
Cholesterol	0.00	18^**∗**^	196^**∗**^

^*∗*^ppm: parts per million; ^*∗∗*^percentage.
